# Use of Both Cumulus Cells’ Transcriptomic Markers and Zona Pellucida Birefringence to Select Developmentally Competent Oocytes in Human Assisted Reproductive Technologies

**DOI:** 10.1186/1471-2164-16-S1-S9

**Published:** 2015-01-15

**Authors:** Mourad ASSIDI, Markus MONTAG, Marc-André SIRARD

**Affiliations:** 1 Center of Excellence in Genomic Medicine Research, King AbdulAziz University, Jeddah, 21589, Saudi Arabia; 2 KACST Technology Innovation Center in Personalized Medicine, King AbdulAziz University, Jeddah, Saudi Arabia; 3 Centre de Recherche en Biologie de la Reproduction, Laval University, Quebec City, QC, G1K 7P4, Canada; 4 Department of Gynecological Endocrinology and Reproductive Medicine, Bonn University, Bonn, Germany; 5 Current address: ilabcomm Gm bH, Eisenachstr. 34; D-53757 St. Augustin; Germany

## Abstract

**Background:**

Selection of the best oocyte for subsequent steps of fertilization and embryo transfer was shown to be the crucial step in human infertility treatment procedure. Oocyte selection using morphological criteria mainly Zona pellucida (ZP) has been the gold standard method in assisted reproductive technologies (ART) clinics, but this selection approach has limitations in terms of accuracy, objectivity and constancy. Recent studies using OMICs-based approaches have allowed the identification of key molecular markers that quantitatively and non-invasively predict the oocyte quality for higher pregnancy rates and efficient infertility treatment. These biomarkers are a valuable reinforcement of the morphological selection criteria widely used in *in vitro* fertilization (IVF) clinics. In this context, this study was designed to investigate the relationship between transcriptomic predictors of oocyte quality found by our group and the conventional morphological parameters of oocyte quality mainly the ZP birefringence.

**Results:**

Microarray data revealed that 48 and 27 differentially expressed candidate genes in cumulus cells (CCs) were respectively overexpressed and underexpressed in the ZGP (Zona Good Pregnant) versus ZBNP (Zona Bad Non Pregnant) groups. More than 70% of previously reported transcriptomic biomarkers of oocyte developmental competence were confirmed in this study. The analysis of possible association between ZP birefringence versus molecular markers approach showed an absence of correlation between them using the current set of markers.

**Conclusions:**

This study suggested a new integrative approach that matches morphological and molecular approaches used to select developmentally competent oocytes able to lead to successful pregnancy and the delivery of healthy baby. For each ZP birefringence score, oocytes displayed a particular CCs' gene expression pattern. However, no correlations were found between the 7 gene biomarkers of oocyte developmental potential and the ZP birefringence score. Further studies using larger lists of candidate markers are required to identify suitable genes that are highly correlated with the morphological criteria, and therefore able to reinforce the accuracy of oocyte selection and the effectiveness of infertility treatment.

## BACKGROUND

Infertility is “a disease” that affects the reproductive apparatus making it unable to carry out a successful clinical pregnancy after at least one year of regular and unprotected intercourse [1]. The worldwide prevalence of this disease keeps rising and is estimated to 1 out of 6 couples of reproductive age. Consequently, an increase need for assisted reproductive technologies (ART) to help couples achieving their parenthood project has been recorded. It is important to highlight that regardless of the infertility cause, the oocyte quality is recognized as the main cornerstone amongst others of any successful ART treatment [2]. Consequently, numerous studies focused on the criteria allowing an efficient selection of high-quality oocytes, named also competent oocytes, for assisted reproduction procedure using both human and animal models. Such oocyte competence is defined as its ability to properly achieve full maturation at nuclear, cytoplasmic and molecular levels, to achieve successful steps of fertilization and early embryo development, and to ultimately lead to a viable and healthy offspring [3-5]. The cumulus-oocyte complex (COC) morphology and cytology are amongst the main parameters that have been used in ART clinics to select the best oocyte for subsequent steps of fertilization and early embryo development in human and livestock species [6,7]. These parameters include the oocyte diameter, its cytoplasm granulation and the first polar body integrity [7,8]. For its bordering cumulus cells (CCs), their appearance, compaction and number of layers have been also correlated to the oocyte developmental potential [9-12]. It is established that the oocyte communication with CCs are a prerequisite to acquire its full developmental competence. In fact, CCs were shown to enhance the oocyte quality by their physical presence (cell-cell adhesion structures), their morphology [12,13], their metabolic and signal transduction functions [14-17].

Zona pellucida (ZP), which is a filamentous matrix of well-structured and glycosylated glycoproteins surrounding the oocyte, is another morphological criterion of oocyte selection. This matrix is made of three proteins encoded by three different genes ZP1, ZP2 and ZP3 [18,19]. An additional protein expressed by a ZP1-paralogue gene, the ZP4, was also reported in human [20-22]. ZP genes’ transcription and glycoprotein synthesis are reported in both the oocyte and CCs of most mammals. This ZP acts as an oocyte’s coat since the oocyte growth and maturation, remains during the early embryo development until the blastocyst hatching [23,24]. Its thickness increases with the oocyte maturation progression to reach around 17 µm at the human MII stage [25]. ZP is also marked by layers of long filaments with repetitive protein heterodimers ZP2-ZP3 cross-linked by ZP1 homodimers [26,27]. ZP proteins’ glycosylation is maximal in the outer layers and decreases progressively to a more compact and less porous inner region [24,28]. In human and mice, ZP3 is crucial for sperm recognition and fertilization [27,29-31]. A positive correlation between ZP Thickness and uniformity, and the human oocyte quality was reported in several studies [9,32-34]. That’s why many human IVF clinics used the ZP thickness and birefringence as a morphological criterion to select oocytes with higher ability to achieve successful pregnancy [32,34-36].

Despite their contribution to a relative improvement of IVF outcomes, these morphological and microscopic-based selection approaches have limitations in terms of accuracy, objectivity and constancy. They rely more on the embryologist/clinician experience since the molecular events underlying the oocyte competence acquisition and their manifestation on its morphology are still poorly understood [37,38]. The alternative of transferring more than one embryo to balance such weakness has led to a significant increase of multiple pregnancies rates, premature births, mother health problems and associated costly health treatments [39-41]. Therefore, the qualitative and quantitative increase of ART outcomes and the reduction of associated health care costs for both the mother and the offspring are still challenging [42,43].

The rapid development of high-throughput OMICs technologies has been a precious adjunct to ART in order to develop more effective strategies to improve the selection of high-quality oocytes using non-invasive and quantitative approaches, allowing therefore more effective elective single embryo transfer (eSET) procedure [44,45]. In this context, some promising strategies based on indirect prediction of oocyte competence/quality using the follicular cells in the oocyte neighborhood mainly CCs were recently suggested. The focus on CCs is mainly due to their continuous bidirectional molecular interplay with the oocyte shown to be crucial for her subsequent developmental competence. Therefore, CCs are thought to reflect the oocyte competence level. Several molecular biomarkers using follicular cells and mainly CCs have been suggested recently by our group [4,46-51] and elsewhere [52-55]. We assume that these molecular biomarkers are a valuable reinforcement to the morphological selection criteria accumulated by the clinicians’ cumulative experience and used in IVF clinics worldwide. In this context, this study was designed to study possible correlations between some molecular predictors of oocyte quality and the conventional morphological parameters of oocyte quality mainly ZP birefringence. We expect to report a positive correlation between the ZP birefringence score and transcriptomic markers of oocyte competence expressed in CCs. Such pioneering approach based on combining the OMICs tools with established oocyte quality parameters mainly the ZP birefringence should lead to an integrative strategy that will accurately predict the oocyte developmental potential. A clinical tool that use both molecular biomarkers and ZP birefringence to efficiently select high quality oocytes in IVF clinics will allow the selection of the best oocyte(s), a successful elective single embryo transfer (eSET) and consequently more effective infertility treatment.

## RESULTS

### Gene expression analysis

RNA hybridizations were performed on a custom-made microarray platform enriched in CCs, granulosa cells (GCs), oocytes and early embryos ESTs using suppressive subtractive hybridization (SSH) of cDNA sequences as described elsewhere [56,57]. Results of microarrays hybridization of CCs of ZGP against those of ZBNP showed that 48 and 27 candidate genes were respectively overexpressed and underexpressed in the ZGP compared to the ZBNP (ZGP/ZBNP ratio ≥ 2; FDR=5%) (see tables S1 and S2, additional files 1 and 2). Interestingly, at least three (3) positive oocyte biomarkers from the (ZGP vs ZGNP) reported in our previous study [49] appeared also in the overexpressed gene list of the (ZGP vs ZBNP). These biomarkers of oocyte quality are PSMD6, CALM1 and NRP1.

### Cumulus cells-induced molecular, cellular and physiological functions

The analysis of the CCs differentially expressed genes (48 overexpressed; 27 underexpressed) (see tables S1 and S2, additional files 1 and 2) showed that several candidates are associated to key functions related to the reproductive function at both molecular and cellular levels (Table 1). Around 23% and 19% of the differentially expressed genes were respectively associated to the reproductive function and inflammatory-like response. These results confirmed the major role of CCs in the final maturation of the oocyte and the ovulation process, known by its inflammatory-like aspect. At the molecular and cellular levels, the time of CCs removal prior to intracytoplasmic sperm injection (ICSI) is marked by an important expansion of extracellular matrix (ECM), active molecular and signaling events to prepare ovulation, steroidogenesis and cell differentiation. That’s why important functions associated to cell morphology (≈ 30% of candidate genes), cell signaling (≈ 17 % of candidate genes) and cellular assembly (≈ 12 % of candidate genes) were activated (Table 1). Conversely, the cell cycle function (≈ 7% of candidate genes) was under-represented since the CCs are in a differentiation stage characterized by more apoptosis rather than proliferation (Table 1).

**Table 1:** Main molecular, cellular and physiological functions triggered by CCs in ZGP versus ZGNP. These functions are ranked by p-value and their proportion of differentially expressed genes (both overexpressed and underexpressed) according to the IPA software.

Molecular, cellular and physiological functions		p-value	No. genes	Proportion of differentially expressed genes involved (%)*
**Physiological functions / conditions**	*Reproductive and embryonic development*	4.13E-05 - 2.95E-02	17	**22.67**
	
	*Organ morphology*	5.92E-06 - 2.95E-02	16	**21.33**
	
	*Inflammatory-like response*	2.86E-04 - 2.95E-02	14	**18.67**

**Molecular & cellular functions**	*Cell Morphology*	5.92E-06 - 2.95E-02	22	**29.33**
	
	*Small molecule biochemistry & signaling*	1.09E-04 - 2.59E-02	13	**17.33**
	
	*Cellular assembly and organization*	4.13E-05 - 2.95E-02	9	**12.00**
	
	*Cell cycle*	1.37E-04 - 2.59E-02	5	**6.67**

*: The proportion of differentially expressed genes included both over- and under-expressed genes

### Real time PCR analysis

**Figure 1:**
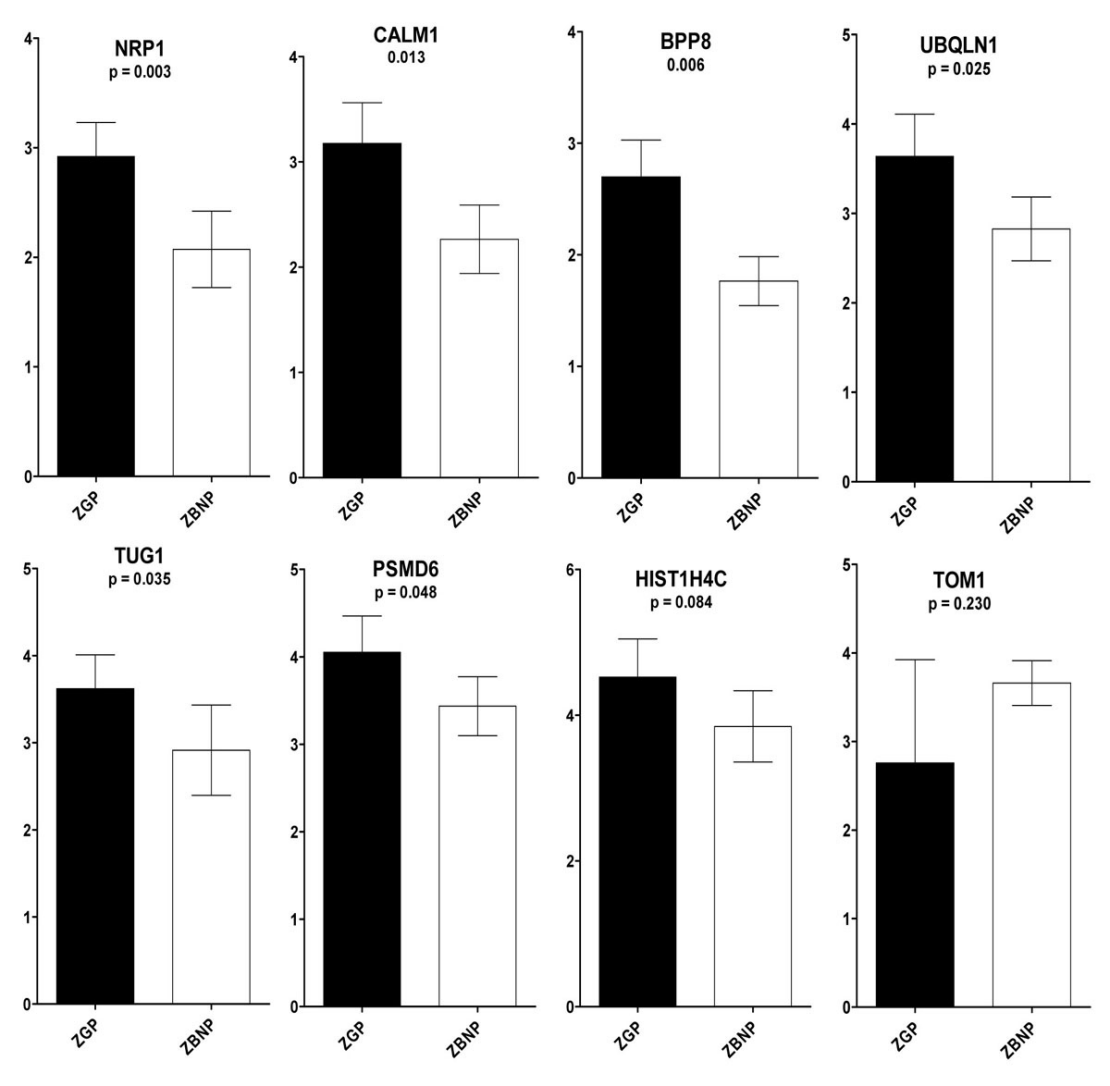
Real-time PCR analysis of differentially expressed genes in individual CCs of ZGP group versus ZBNP. Gene candidates were ranked according to their p-values, which were determined following a T-test analysis achieved on normalized data at α = 0.05

In addition to the validation of the microarray data, quantitative real time PCR was performed on the initial biological samples to assess the expression levels of the 7 gene biomarkers found in our previous study to be overexpressed in ZGP group compared to its ZGNP counterpart [49]. This QPCR validation was achieved on CCs of oocytes with ZGP versus those with ZBNP (successful pregnancy and HZB versus pregnancy failure and LZB). Among the 7 biomarkers associated to both HZB and successful pregnancy reported previously, 5 genes (out of 7) were also significantly different between the two groups studied (ZGP versus the ZBNP) (i.e. 71.4 % of biomarkers confirmed), which is a further quantitative confirmation of the validity of our biomarkers on separate biological samples. These candidates are NRP1 (p = 0.003), CALM1 (p = 0.005), DPP8 (p = 0.006), UBQLN1 (p = 0.025) and PSMD6 (p = 0.048) (Figure 1). However, the association analysis between these biomarkers and ZP birefringence revealed that they were not significantly different between ZGNP and ZBNP (p > 0.05; Table 2).

**Table 2:** Quantitative real-time PCR comparative analysis of differentially expressed genes in individual CCs among the three ZP quality groups (ZGP versus ZGNP versus ZBNP). Candidates with different letters are significantly different following a F-test analysis at α = 0.05

Gene		CALM1	DPP8	HIST1H4C	NRP1	PSMD6	TOM1	UBQLN1
**ZP quality groups**	ZGP	a	a	a	a	a	b	a
	
	ZGNP	b	b	b	b	b	a	b
	
	ZBNP	b	b	a	b	b	a,b	b

### Gene networks’ analysis

**Figure 2:**
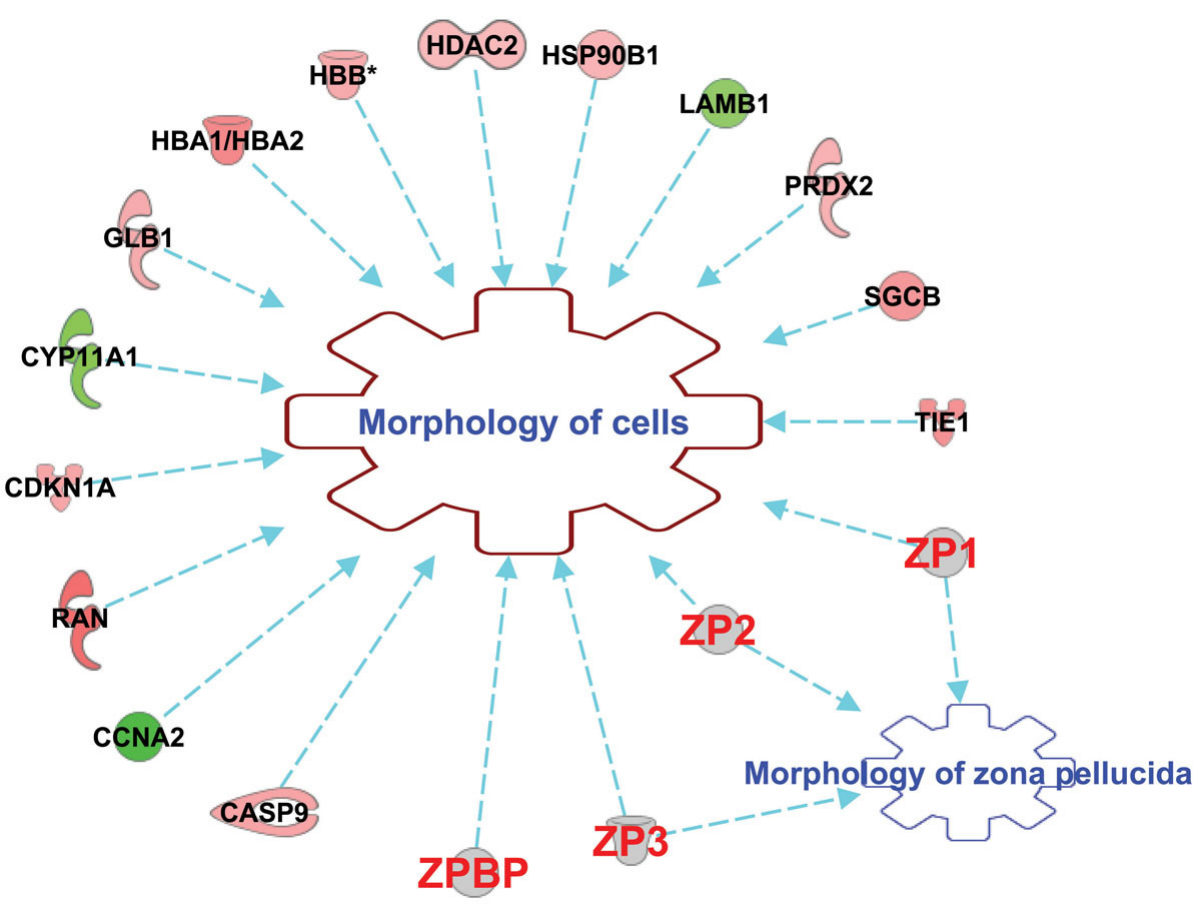
Main candidate genes differentially expressed in CCs and involved in both cell and ZP morphologies as revealed by IPA software. (Red): overexpressed genes. (Green): underexpressed genes. (Gray): ZP genes. Genes are CASP9 (caspase 9), CCNA2 (cyclin A2), RAN (RAN, member RAS oncogene family), CDKN1A (cyclin-dependent kinase inhibitor 1A), CYP11A1 (cytochrome P450, family 11, subfamily A, polypeptide 1), GLB1 (galactosidase, beta 1), HBA1/HBA2 (hemoglobin, alpha 1/ hemoglobin, alpha 2), HBB (hemoglobin, beta), HDAC2 (histone deacetylase 2), HSP90B1 (heat shock protein 90, beta, member 1), LAMB1 (laminin, beta 1), PRDX2 (peroxiredoxin 2), SGCB (sarcoglycan, beta), TIE1 (tyrosine kinase with immunoglobulin-like and EGF-like domains 1), ZP1 (zona pellucida glycoprotein 1 (sperm receptor)), ZP2 (zona pellucida glycoprotein 2 (sperm receptor)), ZP3 (zona pellucida glycoprotein 3 (sperm receptor)), and ZPBP (zona pellucida binding protein).

**Figure 3:**
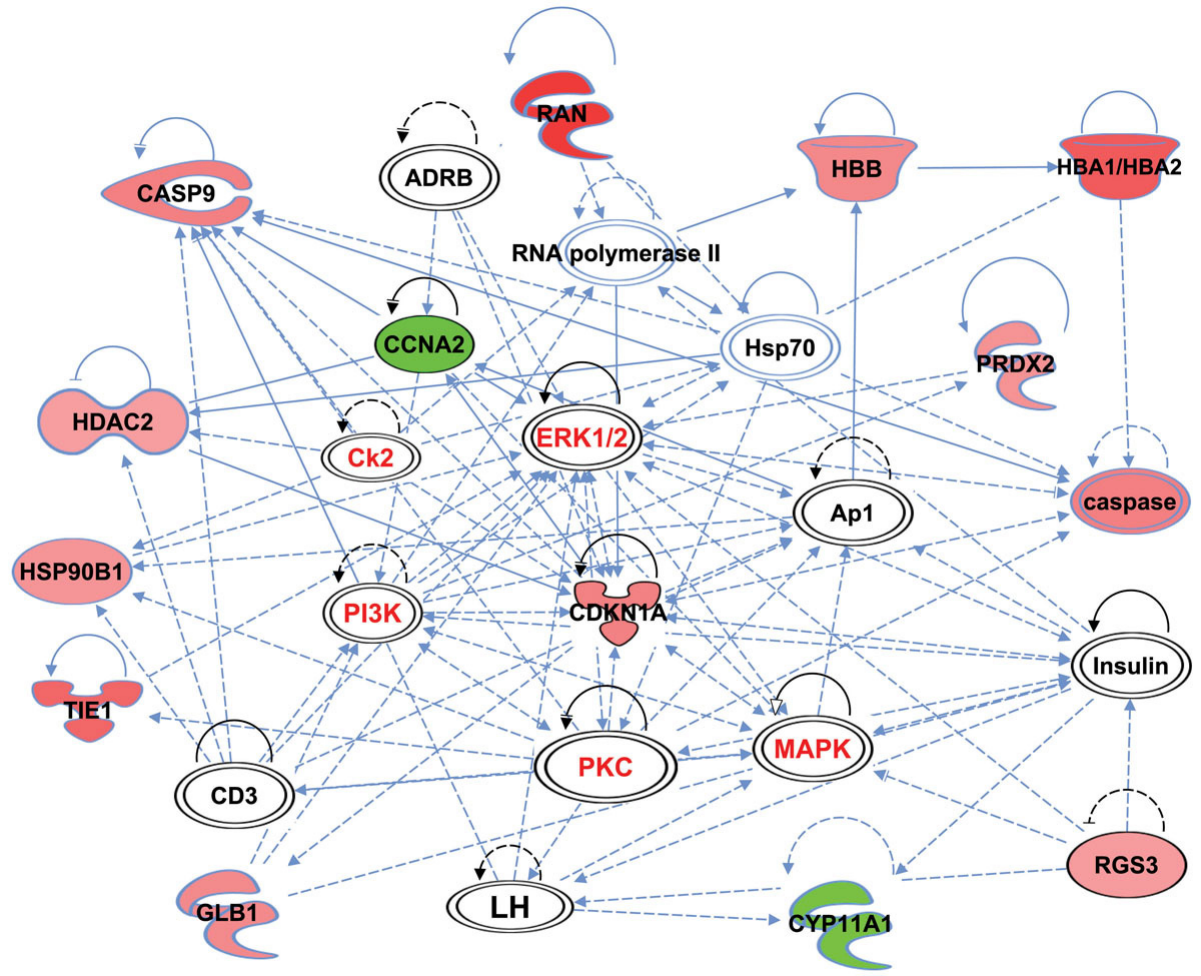
Gene network of differentially expressed candidates in CCs involved in both cell morphology and intracellular signalling pathways cross-talk. (Red): overexpressed genes. (Green): underexpressed genes. (White): other genes. Overexpressed genes are PRDX2 (peroxiredoxin 2), HBA1/HBA2 (hemoglobin, alpha 1/ hemoglobin, alpha 2), HBB (hemoglobin, beta), RAN (RAN, member RAS oncogene family), CASP9 (caspase 9), HDAC2 (histone deacetylase 2), HSP90B1 (heat shock protein 90, beta, member 1), TIE1 (tyrosine kinase with immunoglobulin-like and EGF-like domains 1), GLB1 (galactosidase, beta 1), CDKN1A (cyclin-dependent kinase inhibitor 1A) and RGS3 (regulator of G-protein signaling 3). Underexpressed genes are CCNA2 (cyclin A2) and CYP11A1 (cytochrome P450, family 11, subfamily A, polypeptide 1). Other genes are ADRB (Adrenoreceptor, Beta), Ck2 (casein Kinase II), PI3K (phosphatidylinositol-4,5-bisphosphate 3-kinase), PKC (protein Kinase C), MAPK and ERK1/2 (mitogen-activated protein kinase family), HSP70 (heat shock protein 70), RNA polymerase II, Insulin, AP1 (activator protein-1), CD3 and LH (luteinizing hormone).

**Figure 4:**
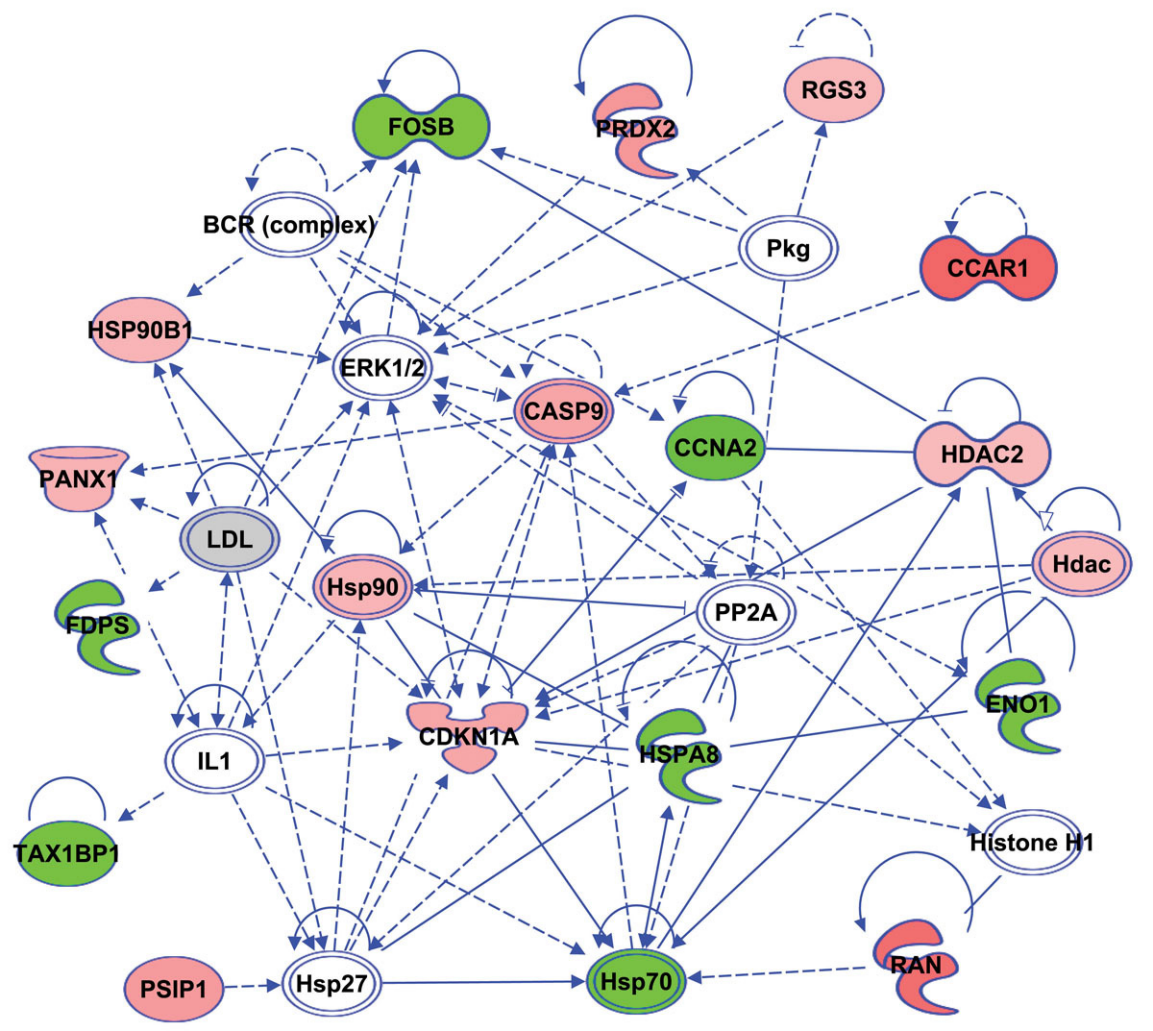
Gene network of several differentially expressed CCs genes involved in both apotosis and anti-inflammatory-like response. (Red): overexpressed genes. (Green): underexpressed genes. (White): other genes. Overexpressed genes are CCAR1 (cell division cycle and apoptosis regulator 1), RGS3 (regulator of G-protein signaling 3), PRDX2 (peroxiredoxin 2), HSP90 / HSP90B1 (heat shock protein 90, beta, member 1), PANX1 (pannexin 1), CASP9 (caspase 9), CDKN1A (cyclin-dependent kinase inhibitor 1A), PSIP1(PC4 and SFRS1 interacting protein 1), RAN (RAN, member RAS oncogene family) and HDAC / HDAC2 (histone deacetylase 2). Underexpressed genes are FOSB (FBJ murine osteosarcoma viral oncogene homolog B), FDPS (farnesyl diphosphate synthetase), TAX1BP1 (Tax1 (human T-cell leukemia virus type I) binding protein 1), HSPA8/ HSP70 (heat shock protein 70 kDa, protein 8), CCNA2 (cyclin A2) and ENO1 (Enolase 1, alpha). The other genes of the network are ERK1/2 (Extracellular signal-regulated kinase 1 /2, mitogen-activated protein kinase family), HSP27 (heat shock 27kDa protein), BCR (complex) (B-cell receptor complex), PP2A (protein phosphatase type 2a), Histone H1, PKG (protein kinase G), LDL (low density lipoprotein) and IL1 (interleukin 1).

Both overexpressed and underexpressed candidate genes of ZGP vs ZBNP groups were mapped to their potential molecular, cellular and biological functions using the Ingenuity Pathways Analysis (IPA) software [58]. Four (4) main networks were selected for further discussion and a comprehensive analysis of differentially expressed genes in CCs collected prior to ICSI and their association with ZP and follicular function. These top-ranked networks were made of differentially over- and under-expressed genes in CCs and focused on: *i*) cell morphology, *ii*) intracellular signaling, *iii*) immune-like response, *iv*) steroidogenesis and *v*) apoptosis (Figures 2, 3, 4).

## DISCUSSION

### Study approach

This study was designed to investigate potential relationships/association between the oocyte quality/competence reflected by its developmental potential and the ZP morphology. For the oocyte competence, it was predicted using transcriptomic markers differentially expressed in its CCs through a comparative analysis of gene expression patterns between ZGP group versus ZGNP in our previous study [49]. Concerning the ZP morphology, it was analyzed using a polarizing microscope (Polscope) linked to a robot-like micromanipulation system (to ensure zona 3D visualisation and scoring) and a suitable software that automatically and objectively (user-independent) measure both ZP density and uniformity. The birefringence score is determined based on approximately 180 measurements that cover all the ZP and was shown to be correlated to the oocyte quality [32,35,59,60]. Taken together, the interesting progress in both ZP birefringence scoring accuracy and the non-invasive and quantitative biomarkers of oocyte quality were behind this original study. The goal is to investigate possible correlations between oocyte developmental competence markers and ZP birefringence in order to assess if these two approaches are additive, and hence able to strengthen each other and enhance the selection of highly competent oocytes procedure. Such positive association once confirmed is expected to improve the infertility treatment approaches through the suggestion of an integrative method based on confirmed molecular predictors that reinforce the morphological selection based on ZP birefringence and the embryologist experience.

### Cumulus cells’ molecular and cellular gene networks

Microarray data analysis revealed a non exhaustive list of 75 differentially expressed genes between ZGP versus ZBNP treatment groups (see tables S1 and S2, additional files 1 and 2) that reflected the behavior and roles of both CCs and ZP prior to ovulation. The analysis of the molecular, cellular and physiological processes triggered by both overexpressed and underexpressed genes revealed key gene networks. The molecular and cellular functions/ networks having the highest scores were selected for further analysis.

#### Cell morphology

It was interesting to report that around 30% of differentially expressed genes in CCs were involved in the regulation of cell morphology (Table 1; Figure 2). These findings showed that important ultrastructural changes occurred in cumulus cells during final maturation and ECM expansion through microtubule and microfilaments organization (RAN, PRDX2), cell differentiation (CDKNA1, HDAC2, GLB1, CCNA2, PRDX2) and intracellular signaling (CDKNA1, TIE1, RGS3) (Figure 3). Similar morphological and metabolic changes in CCs due to the remodeling of their cytoskeleton [61,62], the transzonal projections number and signaling [29,63] and the disruption of CCs shapes and cell-cell contacts [64,65] were also reported. In several mammalian species, it was documented that these CCs intracellular events are essential to support oocyte final maturation, to convey COC some signaling factors and viscoelastic properties that ease its release during ovulation as well as to contribute in sperm recognition and fertilization [66-69]. ZP is also an important structure involved in the organization of its own matrix as well as the oocyte and CCs morphology through dynamic arrangements of its structure and microfilament networks (Figure 2; [25,70]). In this study, it was used as a crucial distinctive parameter between the two treatment groups (ZGP vs ZBNP) which may further explain the upregulation of the cell and organ morphology functions (Table 1).

#### Intracellular signaling

In addition to cell morphology, CCs are characterized by a variety of signaling pathways that finely regulated their functions. Such signaling events are governed by gonadotropins [71-73], the oocyte-secreted factors [74-76] as well as the intra-ovarian endocrine and paracrine environment [77,78]. These signaling pathways involve several players including second messengers’ receptors, transcription factors and kinases as illustrated by figure 3. It is important to note that oocyte competence acquisition and ovulation are still poorly understood and this high cross-talk between key signaling pathways mainly kinases (figure 3) is expected. In fact, CCs is considered as an interface between the oocyte and its surrounding environment through functional signaling cascades involving the pathways of these kinases (ERK, p38MAPK, PKC, PKA and PI3K) known to be crucial for mammalian oocyte quality, CCs expansion, ovulation and even early embryo development [17,78-83].

#### Inflammatory-like response

CCs were retrieved prior to ovulation which is a complex mechanism that allows the rupture of the follicle and the ovarian epithelium to release the COC in the fallopian tube. It is a crucial differentiation step in the reproductive function marked by a local inflammatory reaction that involves different signals from the blood supply in the theca cells as well as steroidogenic and proteases factors produced within the follicle. These signals triggered the CCs expression of many genes associated to an inflammatory-like condition (around 19% of total differentially expressed genes; Table 1) including mainly the underexpression of anti-inflammatory regulators (TAX1BP1, MT-CYB, ENO1) and heat shock proteins (HSPA8, HSP90B1, HSP70); and the overexpression of immune (HLA-DRA, SCARA5, PRDX2) and cell signalling (RGS3, RAN, PSPI1) factors ( (Figure 4, see tables S1 and S2, additional files 1 and 2).

These inflammatory-related genes were suggested as protectors of the oocyte and CCs in an inflammatory and pro-apoptotic environment during ovulation [84,85]. Moreover, this inflammatory-like response is considered as prerequisite for ovulation and subsequent fertilization [84,86-88]. Recent genome-wide association studies (GWAS) showed that CCs gained some immune and neuronal functions required during ovulation in many mammal species including human [3,89,90], mouse [85,91], rat [92] and bovine [56]. Overall, these findings support a significant role of CCs in ovulation.

#### Apoptosis

At this preovulatory period, CCs have reached full expansion marked by weaker cell-cell connections and increased level of apoptosis [93,94]. This apoptotic phenotype was clearly reflected in our study by several differentially expressed genes in CCs and involved in pro-apoptotic pathways as heat shock proteins (HSPA8, HSP90B1, HSP70), caspases (CASP9), cell cycle under-regulation and cell differentiation (FOSB, CCAR1, CCNA2, CDKNA1, HDAC2, PRDX2, PANX1) as well as cell death-related intracellular signaling and transport (FDPS, CDKNA1, RGS3, TAX1BP1, RAN, PSPI1) (Figure 4). Such pro-apoptotic gene pattern was associated to an obvious underexpression of cell cycle genes (less than 7% of differentially expressed candidate genes; Table 1). This apoptosis increase was also reported in follicular cells (cumulus and granulosa) in primates and cattle [6,95,96]. Interestingly, a slight apoptosis in the outer layers of CCs is considered as a morphological criterion that is positively correlated with the oocyte developmental competence [79,93,95,97,98]. Additionally, this apoptotic behavior is expected at this stage since CCs have achieved most of their main roles required to both ovarian function and oocyte quality. We assume that these apoptotic signs are an intrafollicular message about the oocyte preparedness for ovulation or atresia. This apoptotic condition may also amplify the immune and inflammatory-like response that prepares the ovulation process.

#### Steroidogenesis

It is established that follicular growth is governed mainly by gonadotropins and steroids. The nearness onset of ovulation is marked by a fine regulation of steroidogenesis to increase estradiol levels and initiate progesterone synthesis [99,100]. Several genes involved in steroidogenesis (around 23%) were differentially expressed as shown by the top position of the reproductive function (Table 1). This CCs active hormonal activity and early luteinisation was also clearly reflected by several differentially expressed genes in CCs (see tables S1 and S2, additional files 1 and 2) and confirmed in previous reports [95,101-104].

### Zona pellucida and ovarian function

**Figure 5:**
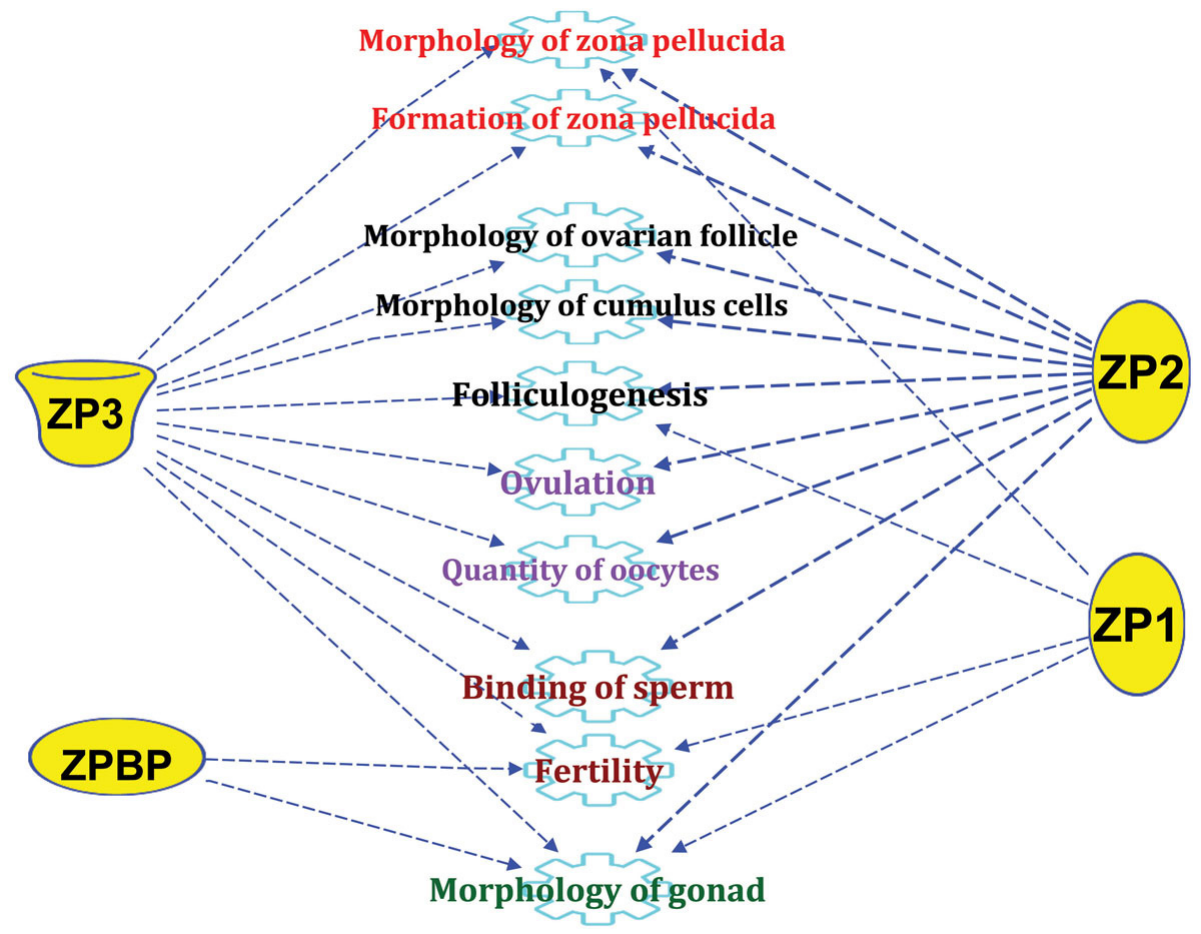
Main ZP genes’ functions related to ovarian function and oocyte quality following a functional analysis using the IPA software. ZP1 (zona pellucida glycoprotein 1 (sperm receptor)), ZP2 (zona pellucida glycoprotein 2 (sperm receptor)), ZP3 (zona pellucida glycoprotein 3 (sperm receptor)), and ZPBP (zona pellucida binding protein).

According to CCs gene expression data, we confirmed herein the complexity of molecular and signalling events that drives the final steps of oocyte maturation, its interplay with CCs and ovulation. Since this study is comparing two treatment groups with different ZP birefringences, it is important to highlight that this oocyte’s coat remain in the oocyte immediate vicinity since the oocyte growth and maturation and remains during the early embryo development until hatching [23,24]. ZP structure is based on filaments of ZP2-ZP3 heterodimers proteins, linked in some binding sites by ZP1[26]. It is involved in vital functions related to ovarian function and oocyte quality as summarized in figures 2 and 5 following gene function analysis using IPA software. In fact, ZP is a filamentous matrix located in the perivitelline space around the oocyte and composed of well-structured and highly- glycosylated glycoproteins [24,28]. ZP genes transcription and their glycoprotein synthesis are reported in both the oocyte and CCs of most mammals and involved in maintaining both COCs and ZP morphologies (Figures 2 and 4), prevent polyspermy and protect early embryo development until the blastocyst hatching [25].

It is important to highlight that according to our gene expression data of this study and elsewhere [49], it appears that each ZP group has its particular gene expression pattern that is dependent on both ZP birefringence and oocyte quality.

## Association ZP birefringence versus oocyte transcriptomic markers

Our data confirmed that CCs compartment is the site of specific signalling and gene expression events which support and reflect the oocyte’s quality. ZP was also showed to be crucial in key functions associated to the oocyte maturation, fertilization and early embryo development. Taken together, CCs and ZP look to be key sites of a well space and time-coordinated sequence of molecular events that determine the oocyte quality and its subsequent developmental competence. The main objective of this study is to study the possibility of combination of these two non-invasive parameters (molecular markers and ZP birefringence) to enhance the effectiveness of high-quality oocyte selection and reinforce the already used morphological criteria. To do this, we have analyzed a list of CCs genomic markers of oocytes having high ZP birefringence (HZB) and were able to achieve successful pregnancy as described in our previous study [49]. Correlation analysis of QPCR results of each one of these transcriptomic markers and both ZP birefringence and pregnancy outcomes revealed that 71.4 % (5 out of 7) of these biomarkers were confirmed using the ZBNP group. This finding is a further quantitative confirmation of the validity of our biomarkers identified on the (ZGP versus ZGNP) study [49] and using separate biological samples. These candidates are NRP1 (p = 0.003), CALM1 (p = 0.005), DPP8 (p = 0.006), UBQLN1 (p = 0.025) and PSMD6 (p = 0.048) (Figure 1). Such results are in line with previous reports confirming the usefulness of CCs transcriptomic markers for selection of the best human oocytes for subsequent fertilization and embryo transfer [42,47,49,98,105], increasing therefore the fertility treatment success rates.

Regarding potential additive effects between these biomarkers and the ZP birefringence, this correlation, if confirmed, could offer an interesting integrative approach based on the combination of morphological (ZP) and molecular (biomarkers) criteria allowing an accurate and non invasive selection of high competent oocytes. Thus, more reliable clinical tools/kits using both parameters could be developed for IVF clinics. However, our data revealed that most of the gene biomarkers were not significantly expressed between the two groups with failed pregnancy (ZGNP vs ZBNP; p> 0.05) except for HIST1H4C (Table 2). Therefore and despite having two opposite ZP birefringence scores (HZB versus LZB), few or no gene biomarkers differences were found between ZGNP versus ZBNP. According to these data, it looks that the ZP morphological phenotype is not directly associated to the selected transcriptomic markers of oocyte competence used in this study. Although the ZP birefringence was positively correlated to oocyte developmental competence, this selection criterion did not revealed significant differences at the gene expression levels once validated by QPCR. As a result, it was difficult to establish a positive correlation/association between the ZP morphology and the molecular marker of oocyte competence expressed in CCs.

Although these data are preliminary and require more investigations, several reasons could explain the lack of correlation between these two parameters. In fact, only 7 gene biomarkers have been used to assess such correlation which might be too small to cover potential genes directly affected by the ZP morphology and/or functions. Larger studies using whole genome microarrays and increased number of selected biomarkers are required to profoundly explore this relationship. Moreover and as mentioned before, the ZP formation started at the secondary follicular stage and is achieved at the preovulatory stage. Therefore at the time of COCs collection for ICSI, low correlation between the ZP birefringence and CCs gene expression may be expected despite their both involvement in subsequent fertilization. Another study by Dr. Montag group [106], reported a differential gene expression gradient within CCs when moving from the very differentiated corona radiata cells (CRCs) at the ZP vicinity to the outer layers. Therefore and due to the high similarities of the compared tissues, only corona CRCs may show a transcriptomic difference associated to ZP birefringence. This assumption is further supported by possible involvement of the CRCs in ZP protein biosynthesis. Therefore, the analysis of the whole CCs in our case may dilute possible transcriptional difference in CRCs with opposite ZP birefringence scores. The possibility that ZP birefringence is an independent parameter that does not unlikely correlate with the genomic predictors of oocyte competence might be also considered.

It is important to highlight that other promising technologies as time-lapse embryo monitoring (TLEM) and preimplantation genetic screening (PGS) can be also combined to our approach [107,108]. Such complementary technologies will even strengthen more our approach through the combination of high quality oocyte selection (suggested herein) and morphologically good quality embryos (TLEM and/or PGS) would offer a very accurate and integrative selection approach that will allow efficient infertility treatments and higher pregnancy successful rates.

## CONCLUSION

Our results confirmed that ZP properties’ variation is associated to a CCs gene expression difference. We also confirmed 5 gene biomarkers of oocyte developmental competence using different biological samples. Surprisingly, no correlations between our 7 CCs gene biomarkers of oocyte developmental potential and the ZP birefringence score. It looks that the ZP morphology is associated to a transcriptomic gene pattern that is not directly related to these biomarkers. These findings highlighted the complexity of the molecular events underlying the developmental competence pathway. Hence, further studies using larger lists of candidate markers are required to identify suitable genes that are highly correlated with the morphological criteria, and therefore able to reinforce the accuracy of oocyte selection. Ultimately, the idea of finding ZP-related biomarkers of oocyte quality would reinforce the accuracy of oocyte selection. Additive or correlated oocyte selection criteria should strengthen each other offering therefore a reliable prognostic tool of the pregnancy outcome. The improvement of oocyte selection procedures would increase the efficiency of infertility treatment as well as the pregnancy outcomes. Additional promising embryo selection technologies as TLEM and PGS are expected to reinforce our current approach through a later selection at the early embryo stage. Therefore, efficient single embryo transfer could be envisioned.

## MATERIALS AND METHODS

### Ethics statement

Patients involved in this study were recruited from the IVF clinic of the University of Bonn Medical School after signing a written informed consent. All procedures and protocols used in this study were approved by the institutional review boards of both the University of Bonn Medical School, Germany; and Laval University, Canada.

### Patients

Seven consenting patients (n=7) were meticulously selected for ovarian stimulation and ICSI (intracytoplasmic sperm injection). Patient selection was based on diagnosis of male factor infertility induced due to low quality sperm (low concentration, morphology or motility) according to World Health Organization (WHO) guidelines [109]. To proper infertility diagnosis and prevent any potential bias, all patients were first subjected to extensive andrological, gynaecological and cytogenetic examinations. ICSI was the recommended procedure for all the patients included in the study. Based on ZP birefringence, CCs of each individual oocyte were divided into two classes: i) HZB: includes 8 CCs of oocytes with High Zona Birefringence; and ii) LZB: includes 3 CCs of oocytes with Low Zona Birefringence.

### IVF and culture media

All media used for oocyte retrieval, denuding, gametes’ washing, ICSI treatment and subsequent embryo culture (Fertilization, Cleavage, Gamete, PVP, Hyaluronidase, Culture oil) were of pharmaceutical, embryo-tested grade and free of phenol red. Unless mentioned, all embryo culture media were provided by Cook Company, Brisbane, Australia.

### Ovarian stimulation and cumulus-oocyte-complexes recovery

A gonadotropin releasing hormone agonist (GnRHa)-based stimulation regimen using triptorelin acetate (Decapeptyl (0.1 mg/day), Ferring, Germany) and HMG (HMG; Menogon, Organon) / FSH (Gonal-F, Serono, Germany) was given to patients as described before [49]. FSH/HMG doses (average of 225 IU) were individually adjusted for each patient using ultrasound monitoring mainly follicular diameter and estradiol. A dose of 10,000 IU of hCG (human chorionic gonadotrophin) was used to induce ovulation and COCs were transvaginally punctured 36 to 38 h later.

### Cumulus cells removal and ZP birefringence assessment

**Figure 6:**
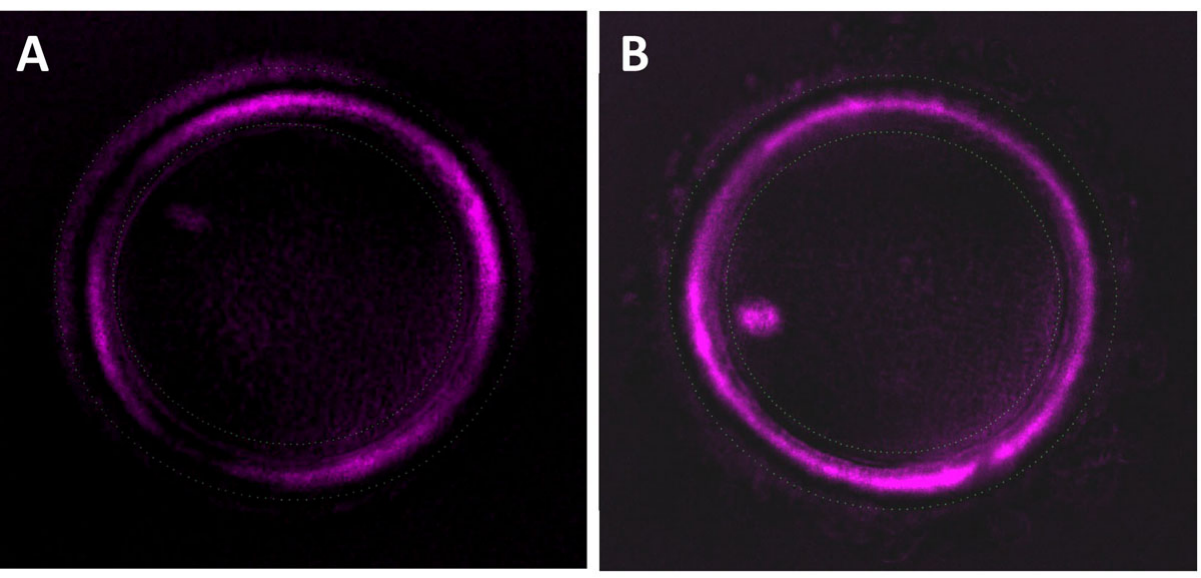
ZP birefringence classes as shown by the polscope. (A): is a low zona birefringence MII oocyte (LZB); (B): is a high zona birefringence MII oocyte (HZB).

Collected COCs were collected, washed and individually incubated at warm temperature using pre-mixed gas with low oxygen (6% CO2, 5% O2, 89% N2) at 37°C. CCs were manually collected using a sterile scalpel in HEPES-buffered medium under oil and put at -80°C. Cumulus-free MII oocytes were selected following a hyaluronidase treatment and thereafter individually incubated in 5-μL droplets of fertilization medium covered with mineral oil at 37°C for subsequent ZP imaging. The analysis of ZP birefringence was automatically done using an automatic module Octax polairAide™ (Octax ICSI Guard™, OCTAX Microscience GmbH, Altdorf, Germany) connected to a polarization imaging software (OCTAX Eyeware™) as reported elsewhere [32]. Zona birefringence visualization and scoring were automatically and non-invasively done at 180 points under a heating platform allowing 37.0 ± 0.5°C in the medium droplet during micromanipulation and microscopic observation. ZP images were captured and used to determine the ZP scores which reflected the uniformity and the intensity of the ZP around the oocyte [32]. While LZB MII oocytes are characterized by an irregular and/or low birefringence distribution, the HZB MII ones had uniform and high intensity birefringence ZP (Figure 6).

### Intracytoplasmic sperm injection (ICSI) and embryo culture

Prior to ICSI, patients were subjected to andrological, gynaecological and cytogenetic examination. The spermatozoa ejaculate was washed, centrifuged, suspended in fertilization media and stored in a CO2 incubator. Following ZP imaging, all MII oocytes with know ZP birefringence and good morphology from patients’ group selected for their male factor-induced infertility received ICSI procedure according to routine standard protocols and using a spermatozoon with good morphology and motility as described elsewhere [49]. Following ICSI, selected zygotes with successful fertilization (presence of two pronuclei (2PN) of equal size in the center of the ooplasm) were individually cultured in a Minc benchtop incubator at 5% O2, 6% CO2, 98% N2 until day 3.

### Embryo transfer (ET) and pregnancy assessment

For each patient and as per law, the two fertilized oocytes with the highest ZP birefringence scores were selected for transfer and the supernumerary oocytes were cryopreserved. The priority for embryo transfer was given to early embryos produced by HZB MII oocytes whenever available; otherwise those coming from LZB ovules were transferred. ET was performed using a transvaginal intrauterine Sydney IVF catheter as described previously [32]. Pregnancy was checked first using a hCG test at day 14 post transfer and confirmed by ultrasounds to detect the presence of gestational sacs and a positive heart beat (viable embryo) at 5 weeks of pregnancy.

### Experimental groups

**Figure 7:**
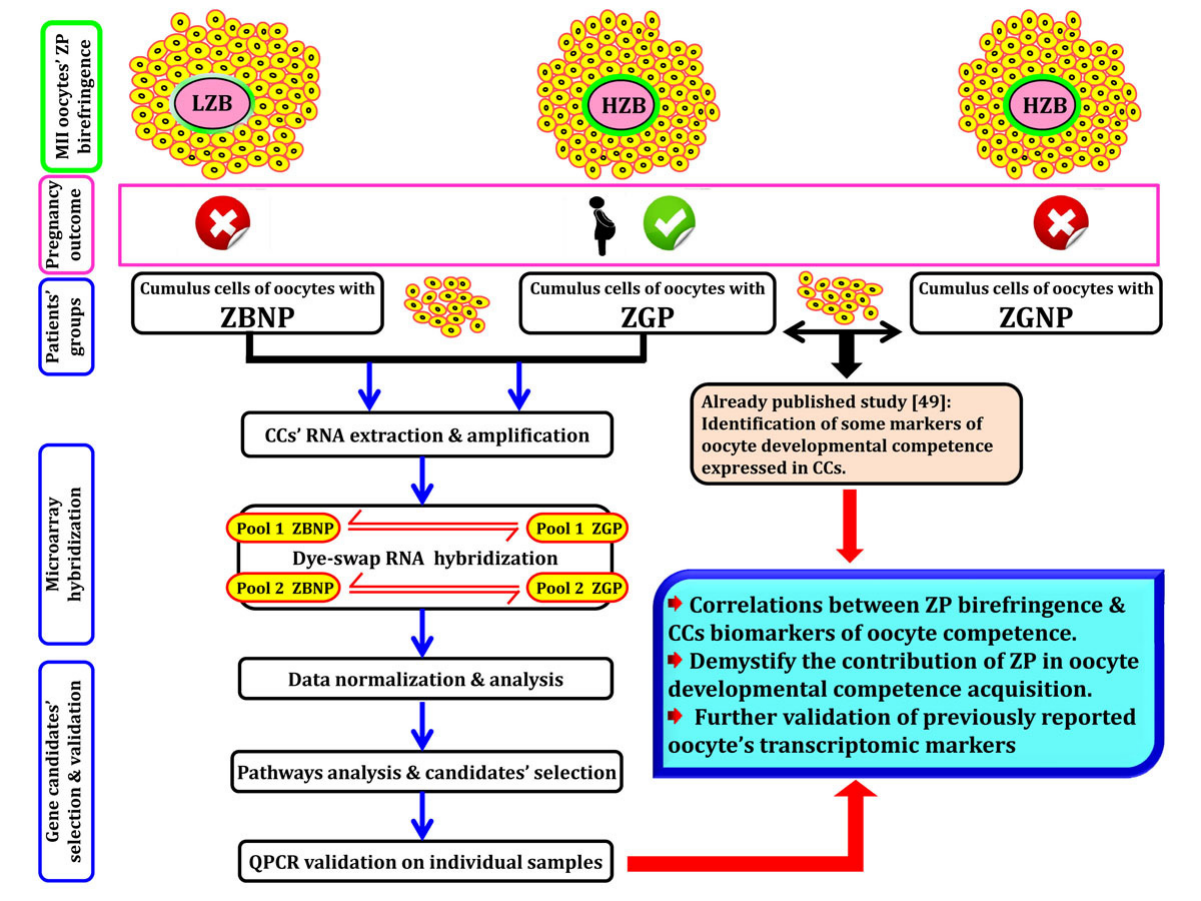
Study’s experimental design

Based on ZP birefringence and subsequent pregnancy results, CCs coming from each individual oocyte were considered as biological replicates and were split into two main experimental groups (Figure 7):

- ZGP (Zona Good Pregnant) group: CCs of HZB oocytes that lead to successful pregnancy.

- ZBNP (Zona Bad Non Pregnant) group: CCs of LZB oocytes and pregnancy failure.

While the ZPG includes 8 CCs and is considered as the positive treatment, the ZBNP one acted as a negative control for ZP relationship with pregnancy.

### RNA extraction and amplification

Total RNA from each CC sample were performed using the PicoPure RNA Isolation Kit (Arcturus, Molecular Devices Analytical Technologies, Sunnyvale, CA) according to the user manual guidelines and eluted in 30 μL of Elution buffer (EB) provided in the kit. Good RNA quality and concentration were assessed and confirmed using the Agilent 2100 bioanalyzer (Agilent Technologies, Waldbronn, Germany) according to the manufacturer’s protocol. For each experimental group, two pools (biological replicates) of 10 ng of total RNA was used for linear amplification of messenger RNA (mRNA) using the 2-round in vitro transcription (IVT) following the instructions of the RiboAmpplus RNA Amplification kit (Arcturus, Molecular Devices Analytical Technologies) as described in the manufacturer's manual. Amplified mRNA yield was eluted in 30 μL of RNA eluted buffer (RE) and quantified by spectrophotometry at 260 nm using the NanoDrop ND-1000 (NanoDrop Technologies, Wilmington, DE).

### Microarray hybridizations

Amplified messenger RNA pools of each group (ZGP vs ZGNP) were labelled using the Universal Linkage System (ULS™) aRNA Fluorescent Labelling Kit (KREATECH Biotechnology, Amsterdam, The Netherlands) according to the manufacturer’s instructions and used in a dye-swap design (Figure 7). Prior to hybridization, an equimolar mixture of the two labelled probes of each ZGP group’s pool and its counterpart in ZGNP group was prepared based on labelling dosage. Hybridization was achieved using a partial custom-made array enriched with transcripts associated to good quality oocytes as described elsewhere [49,57]. Hybridizations were done in the ArrayBooster using the Advacard AC3C (The Gel Company, San Francisco, CA) for 18 h at 50°C using Slide Hyb#1 (Ambion, Austin, TX). The slides were washed successively in (2X SSC/0.5% SDS), (0.5X SSC/0.5% SDS) and 1X SSC buffers, then spin-dried and immediately used for subsequent scanning and analysis.

### Microarray data analysis

Hybridized microarray slides were scanned using the VersArray ChipReader 3.1 System (Bio-Rad, Mississauga, Canada) and analyzed using the ArrayPro Analyzer software (Media Cybernetics, Bethesda, MD). Raw microarray data were first Loess-normalized and corrected for background as described before [56,57]. Ratio of net fluorescence intensities of our dye-swap experiments between positive (ZGP; pregnant) and negative (ZBNP; non-pregnant) groups was assessed using Array Analysis Tool (Baltimore, MD) developed at the National Institute on Aging (NIA) [110] at FDR=5% and a minimum cut-off limit of 2.25. Given that each clone was printed twice on each microarray slide, these two additional technical sub-replicates were considered during data statistical analysis. Two lists of respectively over-expressed and under-expressed gene candidates with more than two-fold change ZGP / ZBNP were generated for subsequent analysis and data validation.

### Gene networks’ analysis

The analysis of gene networks and the molecular pathways triggered by the CCs differentially expressed genes was performed using the QIAGEN’s Ingenuity Pathway Analysis software (IPA) [58]. Briefly, the candidate genes were uploaded with their official name and fold change into the IPA. Such software allowed potential connections and downstream signalling pathways between candidate genes and previous studies to generate and rank causal and/or hierarchical network analysis that may explain the observed gene expression changes in CCs of ZGP vs. ZBNP groups. Such approach was helpful to demystify the molecular pathways and cellular functions associated to ZP birefringence and their potential relationship with the oocyte developmental competence.

### Quantitative real-time PCR of oocyte quality markers

Selected gene candidates mainly the molecular markers of high developmental capacity oocytes reported in a previous study [49] were validated on the original samples of ZGP, ZGNP and ZBNP groups using quantitative real-time PCR. Briefly, equal amounts of total RNA were taken from each replicate on individual CCs of each patient group, denatured and reversed transcribed using the SensiScript reverse transcriptase kit (Qiagen, Mississauga, ON, Canada). Real-time PCR was performed on the selected candidates in LightCycler capillaries (Roche Applied Science, Mannheim, Germany) using the LightCycler FastStart DNA Master SYBR Green I kit (Roche) as well as custom sets of primers reported elsewhere [49] according to the manufacturer’s guidelines. Three housekeeping/control genes ACTB (β-actin), GAPDH, and PPIA were quantified and used in GeNorm Normalization [111,112]. The two housekeeping genes (ACTB and PPIA; P > 0.05) were the most stable and therefore used for QPCR data normalization.

### Association between transcriptomic markers versus ZP birefringence

In order to assess the relationship between the 7 CCs transcriptomic biomarkers of high developmental potential oocytes associated to successful pregnancy and the zona pellucida birefringence, association study between the levels of expression of such biomarkers in 3 patient groups (ZGP vs ZBNP vs ZGNP) having different ZP birefringences and pregnancy outcomes was performed. Therefore and for each gene biomarker, its gene expression levels revealed by QPCR in CCs of the three patients’ groups were compared and analyzed based both the pregnancy output and ZP birefringence score.

### Statistical analysis

Statistical analysis of QPCR results was done by ANOVA followed by Fisher’s protected least significant difference (LSD) test at α=0.05 and using the GraphPad Prism 5 software (GraphPad Software, San Diego, CA). Similarly, association analysis between oocyte quality biomarkers and ZP birefringence was performed for each gene biomarker using the QPCR results of CCs gene expression in the three patients’ groups: ZGP, ZBNP and ZGNP and ANOVA/ F-test as described before.

## ABBREVIATIONS

ZP: Zona pellucida; ART: assisted reproductive technologies; IVF: *in vitro* fertilization; CCs: cumulus cells; COC: cumulus-oocyte complex; MII: metaphase II; eSET: elective single embryo transfer; GCs: granulosa cells; SSH: suppressive subtractive hybridization; ICSI: intracytoplasmic sperm injection; ECM: extracellular matrix; GWAS: genome-wide association studies; CRCs: corona radiata cells; IVT: in vitro transcription; ANOVA: analysis of variance;

## COMPETING INTERESTS

All authors certify that there is no conflict of interest that could inappropriately bias or influence this work.

## AUTHORS' CONTRIBUTIONS

All the experiments have been conducted at Bonn and Laval Universities. Conceived and designed the experiments: MAS, MA and MM. Sample collection: MM. Performed the experiments: MA. Analyzed the data: MA and MAS. Drafted the manuscript: MA. Critical review of the manuscript: MAS and MM. All authors read and approved the final manuscript.

## Supplementary Material

Additional file 1 **Table S1**: List of over-expressed genes in ZGP versus ZBNP groups (ZGP/ZBNP ratio ≥ 2) following microarray analysis at FDR=5% Click here for file

Additional file 2 **Table S2**: List of under-expressed genes in ZGP versus ZBNP groups (ZBNP/ZGP ratio ≥ 2) following microarray analysis at FDR=5% Click here for file

## DECLARATIONS

The publication charges for this article have been funded by the Center of Excellence in Genomic Medicine Research, King Abdulaziz University, Jeddah, Saudi Arabia.

This article has been published as part of *BMC Genomics* Volume 16 Supplement 1, 2015: Selected articles from the 2nd International Genomic Medical Conference (IGMC 2013): Genomics. The full contents of the supplement are available online at http://www.biomedcentral.com/bmcgenomics/supplements/16/S1
