# Supplementary material for: Use of Both Cumulus Cells’ Transcriptomic Markers and Zona Pellucida Birefringence to Select Developmentally Competent Oocytes in Human Assisted Reproductive Technologies
Source: BMC Genomics. 2015 Jan 15;16(Suppl 1):S9. doi: 10.1186/1471-2164-16-S1-S9 (PMC4315169; doi:10.1186/1471-2164-16-S1-S9)
Supplement: Additional file 1 — Table S1: List of over-expressed genes in ZGP versus ZBNP groups (ZGP/ZBNP ratio ≥ 2) following microarray analysis at FDR=5% [file 1471-2164-16-S1-S9-S1.pdf]

Supplemental table 1: List of over-expressed genes in ZGP versus ZBNP groups (ZGP/ZBNP ratio ≥ 2) following microarray analysis at FDR=5%

	Gene	GenBank name	Fold
1	**MOBKL1B**	Homo sapiens MOB1, Mps One Binder kinase activator-like 1B (yeast)	**6.443**
2	**ZNF793**	Homo sapiens zinc finger protein 793 (ZNF793), mRNA	**5.44**
3	**RPL9**	Homo sapiens ribosomal protein L9, mRNA (cDNA clone MGC:87207	**5.267**
4	**GGNBP2**	Bos taurus similar to zinc finger protein 403,transcript variant 2	**5.207**
5	**PSMD6**	Bos taurus proteasome (prosome, macropain) 26S subunit, non-ATPase,	**5.162**
6	**RBMS2**	Homo sapiens RNA binding motif, single stranded interacting protein 2 (RBMS2), mRNA	**4.542**
7	**RAN**	Bos taurus RAN, member RAS oncogene family, mRNA (cDNA clone	**4.535**
8	**KIF13B**	kinesin family member 13B	**4.38**
9	**CCAR1**	Bos taurus cell division cycle and apoptosis regulator 1 (CCAR1),	**4.278**
10	**CALM1**	Homo sapiens calmodulin 1 (phosphorylase kinase, delta) (CALM1	**4.136**
11	**PPM1G**	Homo sapiens protein phosphatase 1G (formerly 2C), magnesium-dependent, gamma isoform	**4.113**
12	**THOC2**	Homo sapiens THO complex 2, mRNA (cDNA clone IMAGE:5556338), partial cds	**3.804**
13	**HBA2**	Bos taurus hemoglobin alpha chain, mRNA (cDNA clone MGC:157272	**3.688**
14	**EIF4GI**	PREDICTED: Bos taurus eukaryotic translation initiation factor eIF4GI protein (EIF4GI), mRNA	**3.453**
15	**FAM73A**	Homo sapiens family with sequence similarity 73, member A (FAM73A),	**3.429**
16	**TIE1**	Homo sapiens tyrosine kinase with immunoglobulin-like and EGF-like domains 1	**3.393**
17	**SYT11**	Bos taurus synaptotagmin XI (SYT11), mRNA	**3.271**
18	**SGCB**	Bos taurus sarcoglycan, beta (43kDa dystrophin-associated glycoprotein) (SGCB), mRNA.	**3.141**
19	**DDX51**	Homo sapiens DEAD (Asp-Glu-Ala-Asp) box polypeptide 51 (DDX51),	**3.116**
20	**PRRT2**	Homo sapiens proline-rich transmembrane protein 2 (PRRT2), mRNA	**3.109**
21	**PSIP1**	Homo sapiens PC4 and SFRS1 interacting protein 1 (PSIP1),	**3.093**
22	**GABARAP**	Homo sapiens GABA(A) receptor-associated protein (GABARAP), mRNA	**3.042**
23	**RDH11**	PREDICTED: Bos taurus retinol dehydrogenase 11 (all-trans/9-cis/11-cis) (RDH11), transcript variant X1	**2.833**
24	**MRPS18B**	PREDICTED: Bos taurus similar to Mitochondrial 28S ribosomal protein S18-2 (MRPS18B),	**2.832**
25	**SCARA5**	Homo sapiens scavenger receptor class A, member 5 (putative) (SCARA5), mRNA	**2.817**
26	**RLIM**	Homo sapiens ring finger protein, LIM domain interacting	**2.784**
27	**CASP9**	Homo sapiens caspase 9 splice variant (CASP9) mRNA, complete	**2.776**
28	**CDKN1A**	Homo sapiens cyclin-dependent kinase inhibitor 1A (p21, Cip1)	**2.744**
29	**VPS16**	Homo sapiens vacuolar protein sorting 16 homolog (S. cerevisiae),	**2.732**
30	**HSD11B2**	Bos taurus 11-beta-hydroxysteroid dehydrogenase type 2 mRNA,	**2.722**
31	**CALM2**	Bos taurus similar to calmodulin 2 (LOC514457)	**2.681**
32	**HBB**	Homo sapiens hemoglobin, beta (HBB), mRNA	**2.604**
33	**GLB1**	Bos taurus GLB1 mRNA for galactosidase, beta 1, complete cds	**2.546**
34	**RPS4X**	Homo sapiens ribosomal protein S4, X-linked (RPS4X), mRNA	**2.519**
35	**PANX1**	Homo sapiens mRNA for pannexin 1 (PANX1 gene),	**2.379**

36	**PRDX2**	Bos taurus peroxiredoxin 2 (PRDX2), mRNA	**2.378**
37	**RNF121**	Homo sapiens ring finger protein 121 (RNF121), transcript variant	**2.36**
38	**HSP90B1**	Bos taurus tumor rejection antigen (gp96) 1 / heat shock protein 90kDa beta (Grp94), member 1	**2.322**
39	**POMP**	Bos taurus proteasome maturation protein (POMP), mRNA	**2.294**
40	**CALU**	Homo sapiens calumein (Calu) mRNA, complete cds	**2.249**
41	**VPS33A**	Homo sapiens vacuolar protein sorting 33 homolog A (S. cerevisiae)	**2.231**
42	**ZFYVE20**	Homo sapiens zinc finger, FYVE domain containing 20, mRNA (cDNA	**2.159**
43	**RGS3**	Bos taurus regulator of G-protein signaling 3 (RGS3), mRNA	**2.15**
44	**RPL37A**	Bos taurus ribosomal protein L37a (RPL37A), mRNA	**2.123**
45	**HDAC2**	Bos taurus histone deacetylase 2 (HDAC2), mRNA	**2.102**
46	**NRP1**	Homo sapiens neuropilin 1 (NRP1)	**2.102**
47	**EMP2**	Bos taurus epithelial membrane protein 2 (EMP2), mRNA	**2.091**
48	**MALAT1**	Homo sapiens MALAT1 mRNA, complete sequence	**2.046**
