# Supplementary material for: Use of Both Cumulus Cells’ Transcriptomic Markers and Zona Pellucida Birefringence to Select Developmentally Competent Oocytes in Human Assisted Reproductive Technologies
Source: BMC Genomics. 2015 Jan 15;16(Suppl 1):S9. doi: 10.1186/1471-2164-16-S1-S9 (PMC4315169; doi:10.1186/1471-2164-16-S1-S9)
Supplement: Additional file 2 — Table S2: List of under-expressed genes in ZGP versus ZBNP groups (ZBNP/ZGP ratio ≥ 2) following microarray analysis at FDR=5% [file 1471-2164-16-S1-S9-S2.pdf]

Supplemental table 2: List of under-expressed genes in ZGP versus ZBNP groups (ZBNP/ZGP ratio ≥ 2) following microarray analysis at FDR=5%

	Gene	GenBank name	Fold
1	SLC25A3	Homo sapiens solute carrier family 25 (mitochondrial carrier;	**3.907**
2	**HNRNPM**	heterogeneous nuclear ribonucleoprotein M	**3.206**
3	**CCNA2**	Homo sapiens cyclin A2, mRNA	**3.087**
4	**ARFGAP3**	Homo sapiens ADP-ribosylation factor GTPase activating protein	**3.018**
5	**RPL10**	Homo sapiens ribosomal protein L10 (RPL10), mRNA	**2.843**
6	**MT-CYB**	Homo sapiens mitochondrially encoded cytochrome b	**2.747**
7	**TAX1BP1**	Homo sapiens Tax1 (human T-cell leukemia virus type I) binding protein 1	**2.712**
8	**HSPA8**	Homo sapiens heat shock 70kDa protein 8 (HSPA8), transcript variant	**2.684**
9	**CYP11A1**	Homo sapiens cytochrome P450, family 11, subfamily A, polypeptide	**2.613**
10	**ENO1**	Homo sapiens mRNA for enolase 1 variant, clone: adSE00169	**2.585**
11	**FDPS**	Human farnesyl pyrophosphate synthetase mRNA, complete cds	**2.567**
12	**FOSB**	Homo sapiens FBJ murine osteosarcoma viral oncogene homolog B	**2.407**
13	**RPL35A**	Homo sapiens ribosomal protein L35a, mRNA (cDNA clone MGC:9770	**2.357**
14	**LAMB1**	Homo sapiens laminin, beta 1 (LAMB1), mRNA	**2.351**
15	**DPH1**	Homo sapiens DPH1 homolog	**2.333**
16	**MT-ATP6**	Homo sapiens ATP synthase 6 mRNA, complete cds; mitochondrial	**2.258**
17	**TNFAIP1**	Homo sapiens tumor necrosis factor, alpha-induced protein 1 (endothelial),	**2.238**
18	**IQWD1**	Homo sapiens IQ motif and WD repeats 1, mRNA (cDNA clone IMAGE:3929120),	**2.214**
19	**TMED5**	Homo sapiens transmembrane emp24 protein transport domain containing	**2.209**
20	**HLA-DRA**	Homo sapiens major histocompatibility complex, class II, DR alpha	**2.187**
21	**RPL14**	Homo sapiens ribosomal protein L14, mRNA (cDNA clone MGC:88594	**2.14**
22	**TTC15**	Homo spaiens tetratricopeptide repeat domain 15	**2.139**
23	**ZNF232**	Homo sapiens mRNA similar to zinc finger protein 232 (cDNA clone	**2.083**
24	**RPS13**	Homo sapiens ribosomal protein S13 (RPS13), mRNA	**2.054**
25	**IFITM3**	Homo sapiens interferon induced transmembrane protein 3 (1-8U)	**2.031**
26	**C20orf4**	Homo sapiens chromosome 20 open reading frame 4 (C20orf4), mRNA	**2.019**
27	**DOM3Z**	Homo sapiens dom-3 homolog Z (C. elegans), mRNA (cDNA clone MGC:29502	**2.006**
